# From the Brazilian Income Transfer Program to the Fiscal Framework: perspectives on the social agenda of Lula’s third Government

**DOI:** 10.1590/0102-311XEN108623

**Published:** 2023-07-17

**Authors:** Daniel Arias Vazquez, Rogerio Schlegel

**Affiliations:** 1 Universidade Federal de São Paulo, Guarulhos, Brasil.

A hallmark of the first and second Luiz Inácio Lula da Silva governments (2003-2006; 2007-2010), the Brazilian Income Transfer Program (*Programa Bolsa Família*, in Portuguese) is analyzed by an article published in this issue of CSP ^1^. The article details the incremental improvements made to the program throughout its existence, and it helps us understand its centrality among social policies. It also discusses disruptions and continuities of cash transfer programs that complemented or succeeded the Brazilian Income Transfer Program - at first under pressure from the acute phase of the COVID-19 pandemic and later against the backdrop of the 2022 election dispute.

The study calls for an update on discussions that have occupied the public debate in recent decades. To what extent do focused policies, aimed primarily at the most vulnerable, represent the best use of public resources to mitigate poverty and inequality? Is the combination of focused and universal social policies the most effective way to ensure equal opportunities ^2^ ^,^ ^3^ ^,^ ^4^ ^,^ ^5^?

The answers to these questions are not isolated from the debate on development strategies for the country. In the previous governments of Lula, social policy was allied to economic policy and vice versa ^6^ ^,^ ^7^ ^,^ ^8^. At the moment that Congress analyzes the so-called Fiscal Framework, with new rules to control public spending, the relationship between the social and the economic returns to the center of the dispute. Lula’s third administration doubles down on Brazilian Income Transfer Program and other social programs, but hints at important concessions to those who believe in an essential rivalry between social policy and macroeconomic policy, as we will discuss below.

Lula’s third Government begins under the sign of reconstruction. If continuity with diversification set the tone for the governments of Dilma Rousseff (2011-2014; 2015-2016) ^9^, the impeachment marked the beginning of a period already characterized as one of policy dismantling, particularly social ones ^10^. Michel Temer (2016-2018) took his fiscal austerity project to the point of constitutionalizing the prohibition of real increases in public spending, creating the Expenditure Ceiling (*Constitutional Amendment n. 95/2016*) ^11^. In addition to promoting underfunding in health and education, the Temer administration bet on the rhetoric that social policies established by the 1988 *Brazilian Federal Constitution*, such as the Continuous Benefit Conveyance (BPC), did not fit in the Brazilian Gross Domestic Product (GDP). Dismantling the BPC was one of the points within a social security reform that, after corruption allegations, Temer did not complete ^12^.

In the Jair Bolsonaro Government (2018-2022), the dismantling of the social protection network was done in the open. Examples of these actions include the cuts in resources and formation of queues for entry into the Brazilian Income Transfer Program, retraction of BRL 20 billion from the Brazilian Unified National Health System (SUS) budget for 2019, end of the policy of real increases to the minimum wage, flattening of the financing of the My Home, My Life program (*Minha Casa, Minha Vida*), and the extinction of the Brazilian National Council for Food Security ^13^. The Social Security reform was complete ^14^. With the onset of the pandemic in 2020, the government was forced to expand the cash transfer - Brazilian Emergency Assistance program (*Auxílio Emergencial*, in Portuguese, reached 67.9 million people ^15^ - and Congress made it possible to break the spending limit, creating a parallel budget to combat COVID-19. For reasons ranging from electoral interests to the resilience of the programs, the income transfer survived the dismantling that reached other areas, not without relevant reconfigurations - such as its denomination as “assistance”, an expression loaded with a welfare character, and the partial demobilization of the technology accumulated in the Single Registry (*Cadastro Único* - CadÚnico) ^16^.

The return of the Brazilian Income Transfer Program was the first act of Lula’s new Government, guaranteed through a strong political articulation still in the transition period. The Brazilian Income Transfer Program currently has more than 21 million families benefited, with an average value of BRL 672 and total transfers exceeding BRL 14 billion, according to the most recent balance sheet of the Federal Government ^17^. If each beneficiary household has four people, we are talking about more than 80 million Brazilians who were reached by the “New Brazilian Income Transfer Program” - a title that the current federal administration uses to underline the idea that, after the Bolsonaro administration, the program was resumed with a new design. The beneficiaries would represent close to 40% of the Brazilian population today. In the current federal administration, complementary benefits were also created, such as BRL 150 for each child up to six years old and BRL 50 additional per pregnant woman.

The Brazilian Income Transfer Program is a conditional transfer of income: cash transfers (not in kind, such as basic food baskets) are made to families (not to individuals), in a focused (they seek to reach the poorest) and conditioned manner (families must fulfill commitments, usually in health and education, to receive their benefits). Until 2017, the program was considered by a study from the Institute of Applied Economic Research (IPEA) as relatively “cheap”, since it obtained relevant effects on poverty and inequality while employing less than 0.5% of GDP. To a large extent, this is due to its good focus: the analysis of the *Brazilian National Household Sample Surveys* (PNADs) from 2001 to 2015 and the Continuous PNADs of 2016 and 2017 shows that this is the transfer, provided by the Federal Government, that most reaches the poorest. Although social security and welfare transfers linked to the minimum wage have a good focus, the Brazilian Income Transfer Program can be even better ^18^.

The advances that the Brazilian Income Transfer Program represented were duly verified by academic production. The program contributed to an increase in food consumption of the benefited families, which presented higher and more significant expenses than the non-benefited ones in a similar situation, according to data from the *Brazilian Household Budget Survey* (POF) of the Brazilian Institute of Geography and Statistics (IBGE) of the late 2000s. It also contributed to increase the percentage of children and adolescents with normal body mass index, indicating greater food security ^19^. On the educational front, a systematic review found that, regardless of the unit of analysis, whether student or school, the Brazilian Income Transfer Program was able to produce positive results, especially for school attendance and dropout rates in the pre-pandemic context ^20^. Municipal economies benefited from the Brazilian Income Transfer Program, considering the association found between benefits paid by the program and the number of people with formal occupation and between benefits and total work income in Brazilian cities ^21^. There are positive impacts on the empowerment of women, who today represent more than 80% of the beneficiaries, observed on different levels, namely on the individual, family, and community levels ^22^. 

Lula’s third term strengthened the Brazilian Income Transfer Program and also recovered other marks of the Worker’s Party (PT) administration in the field of social policies: (1) My Home My Life (Provisional Measure - *MP n. 1162/2023*), with the return of track 1 of the program, intended for families with incomes of up to 2 minimum wages, which admits subsidies of up to 95% of the acquiring property; (2) More Doctors (*Mais Médicos*, in Portuguese; *MP n. 1,165/2023*), with the opening of 15 thousand new vacancies; (3) a real increase of the minimum wage (*MP n. 1,172/2023*), of 3%, after 4 years of freezing, which raised 65% of the benefits paid by the Brazilian National Institute of Social Insurance (INSS), whose value is equal to the floor linked to the minimum wage, including the BPC. Such actions represent important advances in the direction of Brazilian social policy, after an agenda of expenditure cuts ^11^, institutional deconstruction ^14^, and federative discoordination ^23^ in this area during the Temer and Bolsonaro governments.

This resumption of the main social programs of the previous PT governments was made possible by the Transition Constitutional Amendment Proposal (PEC) (*EC n. 126/2022*), which opened a budget space of BRL 145 billion in the 2023 budget, a value very close to the primary deficit forecast this year (BRL 136 billion), corresponding to about 1% of GDP ^24^. That is, the credibility of a newly elected president, added to the ability to sign a broad political agreement, even before his inauguration, allowed the new Lula Government a “license to spend” of an extraordinary nature, valid only for 2023.

This PEC also extinguished the Expenditure Ceiling of the Temer Government, which imposed, in practice, the freezing of federal primary expenditures ^11^, in real terms, for 20 years, which was proved to be unfeasible within the 6 years of its validity. The end of the Expenditure Ceiling was another campaign promise of Lula in the 2022 elections. On the other hand, it would be up to the new government to propose a new fiscal rule by August 2023, to be approved by a National Congress with a more conservative profile than the previous one. It is, therefore, the first major political challenge of the new government, with important implications for the social development strategy of this third term.

In this direction, the third Lula Government elaborated a new Fiscal Framework that, after approval in the Chamber of Deputies, came to be called the New Sustainable Fiscal Regime (Complementary Bill - *PLP n. 93/2023*) and that, at the time of writing this editorial, is being processed in the Federal Senate. The new rules are defined by a double-cap on the growth of federal spending above inflation: (1) up to 70% of the real increase in primary revenues, that is, the 30% portion would be allocated to fiscal adjustment; (2) up to 2.5% of real increase in expenditure, as a maximum limit, having as reference the estimate of potential GDP growth in the medium term, according to the Brazilian Ministry of Finance ^25^. In addition, primary outcome targets were also set for the coming years: to zero the primary deficit in 2024 and generate primary surpluses of 0.5% and 1% of the GDP in the following two years. The real increase in spending is also subordinated to the achievement of these targets, within a tolerance band of ±0.25 percentage points.

On the other hand, this new fiscal arrangement establishes a minimum floor of 0.6% of real increase in federal expenditures, corresponding to the vegetative growth of the population ^25^. That is, only a freeze on spending in per capita values is guaranteed, which does not confer a properly countercyclical character in times of economic downturn. 

Although it is more flexible than the old Expenditure Ceiling, the New Sustainable Fiscal Regime has the same objective of establishing hard budget constraints to ensure a permanent control over state action in the medium and long term. Even if high primary surpluses are generated, as it occurred in the first two Lula administrations, present and future spending will be limited (to the real increase of 2.5% and to 70% of the growth of the collection). These limits will not be exceeded, not even in view of a very positive economic scenario, as to not compromise the fiscal balance in the face of a possible more adverse future conjuncture. This view considers that the Lula and Dilma governments - even under strict fiscal discipline, with triple the primary surplus that planned for now - left a “cursed legacy”: the growth of current expenditures of a social character. The New Sustainable Fiscal Regime is the guarantee to the market that this will not be repeated. 

Furthermore, there is another implicit certainty: under the aegis of this new fiscal framework, the performance of the third Lula Government in the social area will not be the same as the other two mandates. Between 2003 and 2010, federal social spending grew by 70% in real terms, and all areas, without exception, had their resources growing above inflation ^6^. Considering the overall federal primary expenditure, the annual growth in the period was 5.3% - more than double the maximum limit of 2.5% allowed by the new fiscal framework - while social benefits and public investment grew 6.6% and 8.5% per year, respectively ^26^. Concomitantly, there was a strong fiscal adjustment (primary surpluses greater than 3%) and a reduction in the debt/GDP ratio (by about 20 percentage points). 

The assumptions of the new Fiscal Regime seem to commit, to a certain limit, to a rivalry between economic policies and social policies, as it was in the Fernando Herique Cardoso ^27^ Governments and at the beginning of the Lula’s first Government ^28^. It is believed that a permanent fiscal adjustment - with strict controls on the expansion of current expenditure and, consequently, of social spending - is a prerequisite for the reduction of interest rates and that, with this, the pace of growth can be pulled by the private sector, since the growth of the federal public sector will be limited to a lower level. It should be considered that the maximum limit defined by the new fiscal rule will only be reached if economic growth reaches 3.57%, with the tax burden maintained. Under these conditions, the GDP of the private sector will need to accelerate above 4.1% per year, considering the maximum limit of 2.5% of annual growth of public expenditure ^26^. Betting on this profile of economic growth is a very risky strategy because there is less government control over private spending decisions and over the internal and external variables (not only economic, but also political) that affect the “mood” of the market.

The risk of Brazil surrendering to a conflicting vision between the economic and the social will also imply rivalries in the spending decisions of the third Lula Government. In effect, the policy of valuing the minimum wage, the expansion of federal universities, the expansion of health services, the construction of subsidized popular housing, and other important social marks of the PT governments do not fit together in this framework. The increase of one of them by more than 2.5% will imply a lower growth or cuts to other(s), if this maximum level is reached. 

The results obtained in the first and second Lula administrations were able to explain the false dichotomy between the objectives of economic and social policies ^6^ ^,^ ^7^ ^,^ ^8^. In our assessment, the repetition of this good performance in this third term depends less on new fiscal rules and more on a long-term economic and social development strategy, aimed toward the same direction: economic growth, with reduction of inequalities, with the expansion of social policies. We recognize, however, that the terms of this debate are strongly conditioned by the correlation of political forces present within the third Lula Government, considering the fierce electoral dispute, the conservative profile of the National Congress, and the strong influence of economic power on the country’s political-economic agenda.

Since the approval of the New Sustainable Fiscal Regime seems to be inevitable, we can only hope that there will be the expected economic growth, so that we can at least reach the maximum limit of real expansion of social spending. Without high growth (greater than 3.5% per year), we will return inexorably to the recurring debate since the promulgation of the 1988 *Brazilian Federal Constitution* on focused policies versus universal policies ^29^, the decoupling of the minimum wage from Social Security ^30^ and the untying of the revenues constitutionally destined to health and education ^31^, among other proposals for reforms in social policies, guided by the financial market, as new prerequisites for the resumption of economic growth. May we resist again!
